# Determinants of prescribed drug use among pregnant women in Bahir Dar city administration, Northwest Ethiopia: a cross sectional study

**DOI:** 10.1186/1471-2393-14-325

**Published:** 2014-09-18

**Authors:** Chanie Admasie, Belaynew Wasie, Gedefaw Abeje

**Affiliations:** Ethiopian Red Cross Society, Essential drug Program, Bahir Dar, Ethiopia; School of Public Health, College of Medicine and Health Sciences, Bahir Dar University, Bahir Dar, Ethiopia

**Keywords:** Pregnancy, Prescription, Drug use, Bahir Dar city

## Abstract

**Background:**

Drug use during pregnancy may be dangerous to the fetus. There is high consumption of prescribed drugs among pregnant women. This condition may be much higher in developing countries. There is no sufficient evidence on prescribed drug use among pregnant women in Bahir Dar town. The aim of this study was to assess the level of prescribed drug use and associated factors among pregnant women attending antenatal care (ANC) service at government health centers in Bahir Dar city administration.

**Methods:**

Institution based cross sectional study was used. Data were collected from randomly selected 510 pregnant women. Data were analyzed using SPSS version 16.0. Back ward stepwise logistic regression model was used and p-values <0.05 were considered statistically significant.

**Result:**

A total of 510 pregnant women were included in the study of which 88.4% were prescribed at least one drug during pregnancy. Nearly 11% of the pregnant women were prescribed with drugs from category D or X of the US-FDA risk classification.

Prescribed drug use among pregnant women was more likely when the pregnancy is wanted, (AOR = 2.4, 95% CI: 1.3 - 4.6), if the mother had maternal illness (AOR = 8.5, 95% CI: 5.4-13.4), when the educational level of ANC provider is diploma (AOR = 2.7, 95% CI: 1.5-4.7) and when number of pregnancies is more (AOR =2.1, 95% CI: 1.3-3.3).

**Conclusion:**

Prescribed drug use including those with potential harm to the fetus during pregnancy was very high in Bahir Dar city administration. Prescribed drug use is more when the woman had illness, when the woman was multi gravida and when the educational level of ANC provider was low (diploma). It is important to upgrade providers’ educational level and institute prevention of diseases like malaria to reduce the level of prescribed drug use during pregnancy.

## Background

Drug utilization during pregnancy may be unnecessary and dangerous for the fetus [1, 2]. The health care provider must be aware of appropriate pharmacologic therapy for a variety of conditions and the potential impact on pregnant women and their fetus [3, 4]. To guide safe drug use during pregnancy, the U.S. Food and Drug Administration (FDA) classified drugs into five major categories A, B, C, D, and X, with categories D and X indicating evidence of risk in pregnancy [5, 6].

Globally, information on the use of drugs during pregnancy is scarce. Available few studies on the safety and effectiveness of drugs for pregnant women show that physicians prescribe, and pregnant women take a surprisingly large number of drugs. An international investigation done in four continents showed that pregnant women ingest an average of three prescription medications during their pregnancy and 86% of the women had taken at least one prescription medication during their pregnancies [7]. A study done on drug use among pregnant women in Addis Ababa, Ethiopia reported that 71.3% of the pregnant women used at least one drug during their pregnancy. In this study, nearly 4% of the pregnant women were prescribed drugs from category D or X of the US FDA risk classification [8]. The majority of women in Ethiopia live with poor access to health care and the situation of inappropriate drug utilization could be worse. There is no drug prescription guideline for pregnant women in Ethiopia. Data on prescribed drug use among pregnant women in Bahir Dar city administration is lacking. Therefore, this study was conducted to assess the level of prescribed drugs use among pregnant women in Bahir Dar city administration and identify factors associated with prescribed drug use.

## Methods

The study was conducted in Bahir Dar city administration, Northwestern part of Ethiopia. The projected population of Bahir Dar city administration was 239,721 for the year 2012 [9].

Institution based cross-sectional study was conducted from June 20 – July 10, 2013 in government health centers. All pregnant women who came for ANC services to the selected health centers during the study period were study populations.

Pregnant women at any gestational age who were following the ANC service at the selected health centers were included in the study. But pregnant women who were referred from other health institutions outside Bahir Dar city administration were excluded from the study. Pregnant mothers who came more than once during the study period were interviewed only once at their first visit. A woman is said to have used prescribed drug if she reported using prescribed drug during her current pregnancy.

Sample size was determined using single population proportion formula [10] considering proportion of drug use during pregnancy 71.3% [8], 5% margin of error and design effect of 1.5. After adding 10% non-response rate, the final sample size was 518. Multistage random sampling method was used to select the required number of pregnant mothers. Health centers were stratified as rural and urban. Then, two rural and three urban health centers were selected by lottery method. Finally, proportional numbers of pregnant women were taken from each health center based on the flow of pregnant women per day calculated from previous month’s ANC record. Systematic random sampling method was used to select the pregnant women in each health facility.

Data were collected by semi-structured questionnaires and reviews of antenatal follow up cards of pregnant women. First, women were interviewed when she was at waiting room. Then exit interview was done to know the type of drug she was prescribed. Finally, her ANC chart was reviewed to know drugs prescribed previously. Nurses who worked in ANC rooms in other health centers and who had data collection experience collected the data.

The data extraction forms were used to collect information on the total number of ANC visits, gestational age and drugs prescribed during each trimester. The semi-structured questionnaire was used to collect socio-demographic data, obstetric and medical history of pregnant women.

Data were entered in to Epi-info 3.5.2 and analyzed using SPSS version 16.0. Bivarate analyses were performed and variables with p < 0.2 were included in multivariable logistic regression using backward stepwise logistic regression model. Hosmer and Lemeshow goodness of fit test was used to check model significance and p < 0.05 was used to determine statistical significance.

Ethical clearance was obtained from Research Ethics Review Committee of Bahir Dar University. Permission to conduct the study was obtained from Amhara Region Health Bureau, Bahir Dar city health office and the respective health centers. Data collection was conducted after explaining the purpose of the study to the participants and obtaining informed verbal consent.

## Results

A total of 510 (response rate of 98.5%) pregnant women were studied.

### Socio demographic characteristics

The majority of the respondents (77.5%) were in the age group of 20 and 34. The mean (±SD) age of women was 26.5 (±6.0) years. Four hundred sixty eight (91.8%) respondents were married and 40(7.8%) were single.

Regarding their educational status, 151(29.6%) women were unable to read and write, while 37 (7.3%) of them were able to read and write. One hundred eight (21.2%) completed primary education, 103(20.2%) had completed secondary school, while 111 (21.8%) had attended higher level education (Table 1).

**Table 1:** **Socio-demographic characteristics of pregnant women attending ANC Service in Bahir Dar city administration, June 20 - July 10, 2013, Northwest Ethiopia**

Variable	Number of women	Percent
**Age in years**		
≤ 19	49	9.6
20-34	395	77.5
35 – 42	66	12.9
**Occupation**		
Government employed	92	18
NGO employed	49	9.6
Merchant	97	19
House wife	210	41.2
Student	38	7.5
Unemployed	17	3.3
Others^*^	7	1.4
**Monthly income(in birr)**		
< 800.00	118	23.1
801 – 1000.00	151	29.6
1001- 2000.00	168	32.9
>2000.00	73	14.3

^*^House servant and daily laborer.

### Obstetric and medical history

Sixty five (12.7%) of the pregnant women reported history of chronic disease. More than half of the pregnant women (54.3%) were primigravida and the rest 233(45.7%) were multi-gravida. Three hundred sixty two (71%) of the pregnancies were wanted, 78(15.3%) were mistimed and 70 (13.7%) were unwanted pregnancies. Forty three (8.4%) respondents reported history of hospitalization during their current pregnancy. Three hundred ninety nine (78.2%) of the respondents had 1–2 total ANC visits, and 111 (21.8%) of them had 3–4 total ANC visits on the date of interview.

### Drug use during pregnancy

A total of 451(88.4%) pregnant women were prescribed at least one drug during their current pregnancy. Among these, three hundred fifteen (61.8%) were prescribed at least one drug excluding iron. The mean number of drugs prescribed during pregnancy was 2.3. For the current pregnancy, 103(22.8%), 166(36.8%), 128(28.4%), 43(9.5%), 8(1.8%) and 3(0.7%) women were prescribed one, two, three, four, five and six drugs respectively. Thirty three different types of drugs (a total of 1049) were prescribed for the respondents.

**Figure 1:**
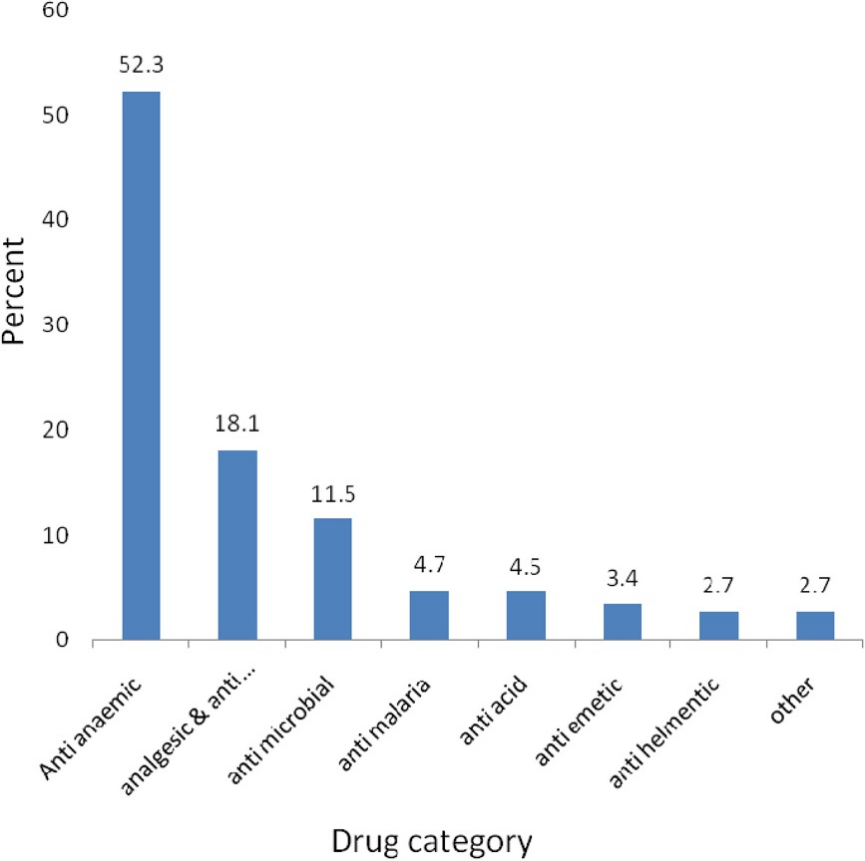
**Percentage of commonly prescribed class of drugs to pregnant women attending ANC service in Bahir Dar city administration, June 20-July10, 2013 Ethiopia.** Others include: - antispasmodics, laxatives, anti-protozoa, vitamins cough syrups, ant-hypertensive and anti-asthmatics.

**Figure 2:**
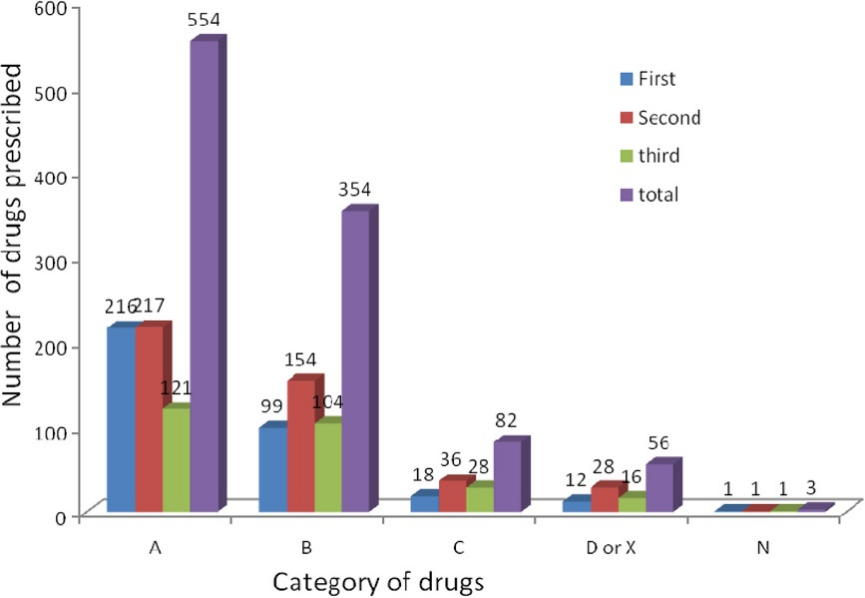
**Category of drugs prescribed according to US FDA risk category and gestational age among pregnant women attending ANC service in Bahir Dar city administration June 20-July10, 2013, Ethiopia.**

The most commonly prescribed classes of drugs during first trimester of pregnancy were anti-anemic. Considering all trimesters, the most commonly prescribed and recorded class of drugs during pregnancy was anti-anemic preparations (see Figures 1 and 2).

**Figure 3:**
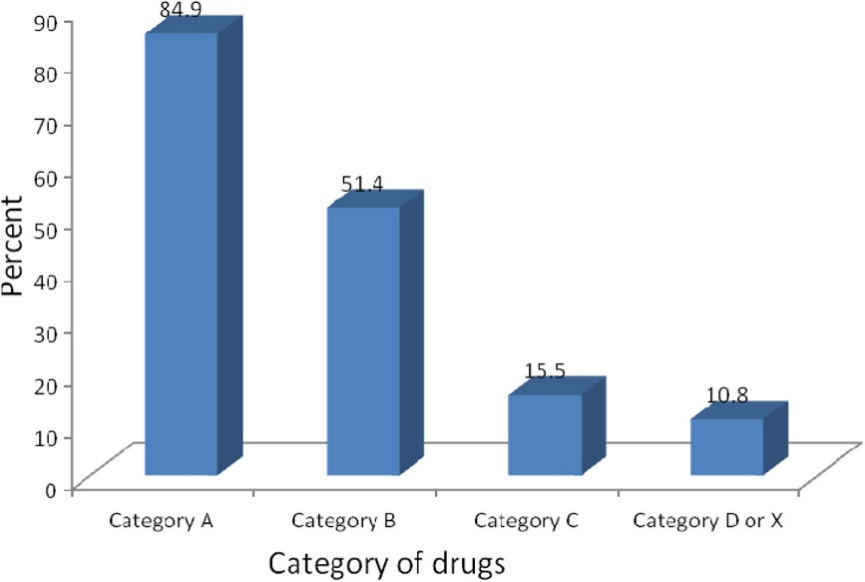
**Percentage of pregnant women exposed to drugs according to US FDA risk category among pregnant women attending ANC service in Bahir Dar city administration June 20-July10, 2013 GC, Ethiopia.**

The most frequently prescribed category of drugs during the first trimester were US FDA category A drugs. Similarly, during the second trimesters, US FDA category B drugs were the most frequently prescribed drugs. Considering all trimesters of pregnancy, US FDA category A drugs were the most frequently prescribed. The frequently prescribed drugs include iron/fefol, from US FDA category A and paracetamol and amoxicillin from US FDA category B drugs. Fifty six (5.3%) of all the prescribed drugs were from US FDA category D. The proportion of pregnant women prescribed a US FDA category D or X drug were 10.8%. US FDA Category D or X drugs were prescribed during all trimester of pregnancy with the highest frequency during the second trimester. Drugs in US FDA category D or X which were commonly prescribed include quinine and co-trimoxazole (trimethoprim + sulphamethoxazole) (Figures 2 and 3).

### Factors associated with prescribed drug use during pregnancy

Multivariable logistic regression model revealed that only pregnancy status, maternal illness on the date of interview, educational level of provider and number of gravida were significantly associated with prescribed drug use during pregnancy.

Multi gravida women were 2.1 (AOR = 2.1, 95% CI: 1.3-3.3) times more likely to get drugs prescribed compared to primigravida women. Pregnant women with current illness were 8.5 (AOR = 8.5, 95% CI: 5.4-13.4) times more likely to get a drug prescribed than those who had no current maternal illness. Pregnant women with wanted pregnancy were 2.4 (AOR 2.4, 95% CI: 1.3 -4.6) times more likely to get a drug prescribed than those with unwanted pregnancies. Similarly, pregnant women who were provided ANC by diploma holders were 2.7 (AOR = 2.7, 95% CI: 1.5-4.7) times more likely to get a drug prescribed compared to those women who were provided ANC by degree holders (Table 2).

**Table 2:** **Factors associated with prescribed drug use during pregnancy among pregnant women attending ANC service in Bahir Dar city administration Northwest Ethiopia, June 20-July10, 2013**

Variable	Drugs prescribed		COR, 95% CI	AOR, 95% CI	P-value
	Yes	No			
**Age of women in years**					
15-19	22	27	1	1	
20-34	240	155	1.9(1.1 – 3.5)	1.1(0.5-2.4)	0.819
35-42	53	13	5(2.2-11.5)	0.9(0.3-2.9)	0.941
**Educational level of women**					
No formal education	137	51	2.2(1.5-3.2)	1.2(0.7-2.0)	0.471
Attained formal education	178	144	1	1	
**Monthly income (in birr)**					
100–800	103	55	1.4(0.9-2.2)	1.2(0.7-2.2)	0.550
801–1000	75	36	1.6(0.9-2.5)	0.9(0.5-1.7)	0.797
>1001	137	104	1	1	
**Gravidity**					
1	235	42	1	1	0.002
>1	216	17	2.3(1.3 – 4.1)	2.1(1.3-3.3)	
**Pregnancy status**					
Wanted	228	134	2.0 (1.2 -3.4 )	2.4(1.3 -4.6 )	0.006
Mistimed	55	23	2.8(1.4 – 5.6)	2.1(0.9 -4.9)	0.093
Unwanted	32	38	1	1	
**Current maternal illness**					
No	91	156	1	1	
Yes	224	39	9.8(6.4-15.1)	8.5(5.4-13.4)	<0.001
**Educational level of provider**					
Degree	30	59	1	1	
Diploma	285	139	4.1(2.5-6.7)	2.7(1.5-4.7)	0.001
**Hospitalization history**					
No	278	189	1	1	
Yes	37	6	4.2(1.7-10.1)	2.6(0.9-7.1)	0.060
**Number of ANC visits**					
1-2	239	160	1	1	
3-4	76	35	1.5(0.9-2.3)	1.7(0.9-2.9)	0.055
**Location of health facility**					
Urban	222	161	1	1	
Rural	93	34	2(1.3-3.1)	1.5(0.9-2.7)	0.140

## Discussion

In this study, a total of 451(88.4%) pregnant women were prescribed with at least one drug during their current pregnancy. Excluding iron only prescription, 315(61.8%) of the pregnant women were prescribed at least one drug. A total of 56(10.8%) pregnant women were prescribed drugs from US FDA category D or X.

In this study, the proportion of pregnant women who were prescribed drugs (88.4%) was higher than a study done in Addis Ababa which revealed that 71.3% of the respondents took at least one drug during their pregnancy [8]. This relatively higher extent of drug use may be due to the implementation of iron/fefol supplementation policy. This finding was similar to studies in Norway, Sweden (86%) and Germany (85%) [11, 12] and lower than a study in Hungary (92%) and Brazil (94.9%) [2, 13].

The most commonly prescribed drugs to pregnant women in this study were anti-anemic followed by analgesic, anti-microbial, anti-malaria and antacids. This was consistent with the study done in Addis Ababa and Pakistan [8, 14]. The reason is that anemia, head ache and gastritis are the common physiologic problems during pregnancy.

This study indicated that US FDA category A drugs were the most frequently prescribed drugs during pregnancy followed by US FDA category B, C, D or X. The proportion of women prescribed with US FDA risk category D drug during their pregnancy in this study was two times higher than findings in US, Pakistan and Addis Ababa. A study in USA using data from eight health maintenance organizations showed that 4.8% of pregnant women received a drug from category D [15]. Another study conducted to evaluate patterns of drug prescriptions to pregnant women in tertiary care hospitals in Pakistan showed that 2.3% of drugs prescribed to pregnant women were considered to be teratogenic and twenty nine pregnant women (0.8%) were prescribed these teratogenic drugs [14]. Similarly a study done in Addis Ababa indicated that nearly 4% of pregnant women were prescribed from category D or X [8].

The proportion of pregnant women prescribed US FDA category D or X drugs were 10.8% which is not comparable to the findings of a study conducted in Addis Ababa (8). But this finding was comparable with a study done in Nigeria, which showed that 13% of pregnant mothers were prescribed from category D [16].

Among the 56 drugs prescribed from US FDA category D or X drugs, 49(87.5%) were quinine tablets prescribed to treat malaria. This is high compared to finding of a study in Addis Ababa. The reason for this difference is high prevalence of malaria in Bahir Dar city administration. The other US FDA category D drugs prescribed to pregnant women include doxycycline and co-trimoxazole (trimethoprim and sulphamethoxazole combination). Among the category D drugs prescribed in this study, co-trimoxazole was mentioned in a study done in Addis Ababa [8].

Our findings indicated that there is significant association between drugs prescribed during pregnancy and gravidity, maternal illness on the date of interview, pregnancy status and educational level of the provider. These findings are comparable to other studies [2, 8, 17]. The reason for this may be as number of pregnancy increases, risk of maternal illness increases. Similarly, as educational level of the provider increases, the probability of prescribing many drugs will decrease.

There was a possibility of recall bias while trying to gather data on previous medical and drug use history. This was minimized through the use of multiple data sources as interview, and medical records to check for the previous drugs used.

## Conclusions

The level of prescribed drug use including those with potential harm to the fetus during pregnancy was very high in Bahir Dar city administration. The prescribed drug use during pregnancy was higher when the pregnancy was wanted, if the mother had illness on the date of interview, when the educational level of provider is low (diploma) and when the woman is multi gravida. Therefore, it is essential to upgrade health professionals who are providing ANC service from Diploma to Degree level to reduce the risk of prescribing unnecessary drugs to pregnant women. Malaria prevention methods should be also strengthened as anti malarial drugs are the most frequently prescribed category D or X drugs to the pregnant women in this study. Food, medicine and health care administration and control authority of Ethiopia should develop guidelines that guide drug prescription for pregnant women. We also recommend further study to identify factors associated with category D or X drug prescription with larger sample size.

## Authors’ information

Mr Chanie is bachelor of pharmacy working in Ethiopian Red Cross society essential drug program, Bahir Dar. Dr Belaynew is an Associate Professor of public health. He is currently working in Bahir Dar University College of medicine and health sciences. Mr Gedefaw is also working in Bahir Dar University College of medicine and health sciences. He is lecturer in the department of reproductive health. He studied nursing and master of public health in Reproductive Health.
